# 

**Published:** 1902-09-15

**Authors:** 


### ANNOUNCEMENTS.

SECOND annual meeting of the American Society of Orthodontists, Philadelphia, October 8, 9, 10, 1902, headquarters, Continental Hotel.

PAPERS.—The President's Address; Normal and Pathological Anatomy of the Alveolar Process and Adjacent Tissue, M. H. Cryer; Art in Relation to Orthodontia, Edward H. Angle; The Deformities of the Superior Maxilla from the standpoint of the Rhinologist, C. H. Kohler; The Causes of Malocclusion, Wm. J. Brady; Retrusion of Both Jaws with a Single Appliance, R. Ottolengui; Nasal Occlusion and Septal Deviation in their Relation to Antrul Development and Facial Expression, Royal S. Copeland; Orthodontia from the Standpoint of a Student, Anna Hopkins; Distal Movement of Molars and Bicuspids Limiting Extraction, Lloyd S. Lourie; Stationary and Removable Appliances alone and in Combination, H. A. Pullen; Subjects to be announced, W. Booth Pearsall, J. Humphries, J. E. Grevers, E. C. Kirk.

Time will be reserved for the consideration of specimens pertaining to orthodontia, which any one may care to present. A cordial invitation is extended to the profession. MILTON T. WATSON, *Secretary*.

270 Woodward Avenue, Detroit.

THE semi-annual meeting of the Central Michigan Dental Association will be held at Ionia, October 1st and 2d, next. P. L. CAMBELL, *Secretary*.

THE next meeting of the South-western Michigan Dental Society, will be held at Three Rivers, September 9th and 10th. C. W. JOHNSON, *Secretary*.

